# A Rare Complication of Feeding Jejunostomy: Murky Waters of the Surgeons

**DOI:** 10.7759/cureus.12945

**Published:** 2021-01-27

**Authors:** Siddhi Chawla, Binit Sureka, Vaibhav K Varshney, Raghav Nayar, Kelu S Sreesanth

**Affiliations:** 1 Diagnostic & Interventional Radiology, All India Institute of Medical Sciences, Jodhpur, Jodhpur, IND; 2 Surgical Gastroenterology, All India Institute of Medical Sciences, Jodhpur, Jodhpur, IND

**Keywords:** feeding jejunostomy, intussusception, complication

## Abstract

Feeding jejunostomy (FJ) is a common surgical procedure for patients presenting with absolute dysphagia. Jejunostomy tube-induced intussusception is an extremely rare complication associated with it and its recognition and proper management are necessary to prevent subsequent bowel ischemia of the intussusception. We present a rare case with simultaneous intussusception at two sites in a patient who underwent FJ with Foley’s catheter one month back and subsequently managed by surgical reduction and repositioning of the FJ tube.

## Introduction

Jejunostomy is the procedure by which a tube is placed in the lumen of proximal jejunum to administer nutrition in patients who cannot take orally due to diseases affecting oesophagus, stomach, duodenum, pancreas, liver or biliary system. The complications seen with jejunostomy can be mechanical, infectious, gastrointestinal or metabolic [1]. Among the various complications, jejunostomy tube-induced intussusception (JTI) is very rare and has an incidence of one per cent. It may be transient, intermittent or non-reducible. The patient usually presents with vomiting, persistent pain or mass per abdomen [2]. We present a case with double intussusception that has not been reported previously and also it is the subsequent surgical management.

## Case presentation

 

An 18-year-old female had a history of caustic ingestion secondary to which she was suffering from dysphagia due to thoracic oesophageal stricture and pyloric stenosis. Subsequently, she underwent an open feeding jejunostomy (FJ) with the use of Foley’s catheter to give high protein diet to build her for definitive surgical intervention. She presented after one month with failure to gain weight and sudden onset pain in that abdomen for two days.

**Figure 1:**
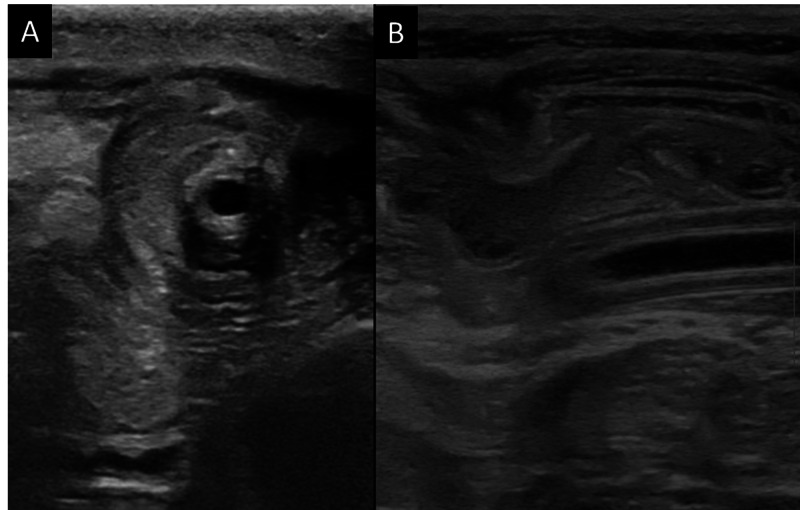
(A) Ultrasound transverse images showing “target” or “bowel within bowel” or “doughnut” appearance of bowel classical for intussusception. (B) Longitudinal scan shows “sandwich” appearance with tube as the leading point.

**Figure 2:**
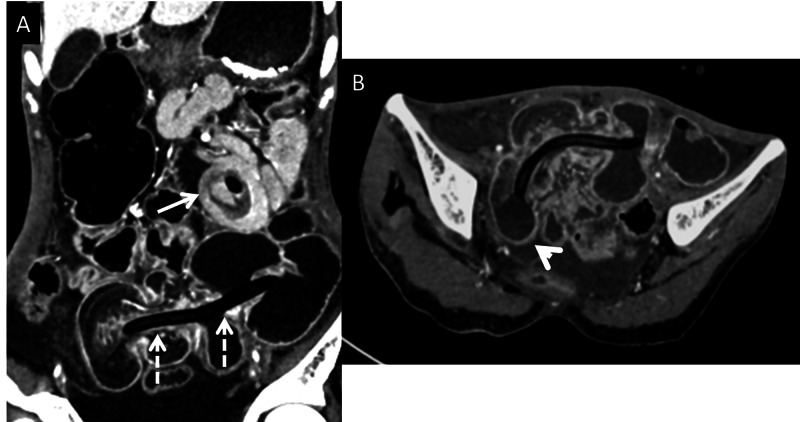
CECT images: (A) Coronal sections showing the proximal (arrow) intussusception site as “target" sign and entire extent of distal intussusception (dashed arrows) with hypoenhancing oedematous inner bowel wall and constricted at entry site. (B) Axial image confirming the Foley’s bulb (arrowhead) as the lead point for the distal intussusception site.

Ultrasound was done which showed “bowel within bowel appearance” and “target sign” in the right lower quadrant of abdomen around the FJ tube suggesting intussusception (Figure 1). Contrast-enhanced computed tomography (CECT) was requested by the clinician in order to confirm the diagnosis. CECT confirmed the diagnosis of intussusception in the distal bowel. However, it also demonstrated a second intussusception near the entry point of the tube. The distal intussusception was identified secondary to the hyper-inflated bulb of the Foley considered a probable lead point (Figure 2). Since the possibility of non-surgical reduction of intussusception was minimal and subsequent bowel ischemia could develop, the patient was taken up for emergent laparotomy.

**Figure 3:**
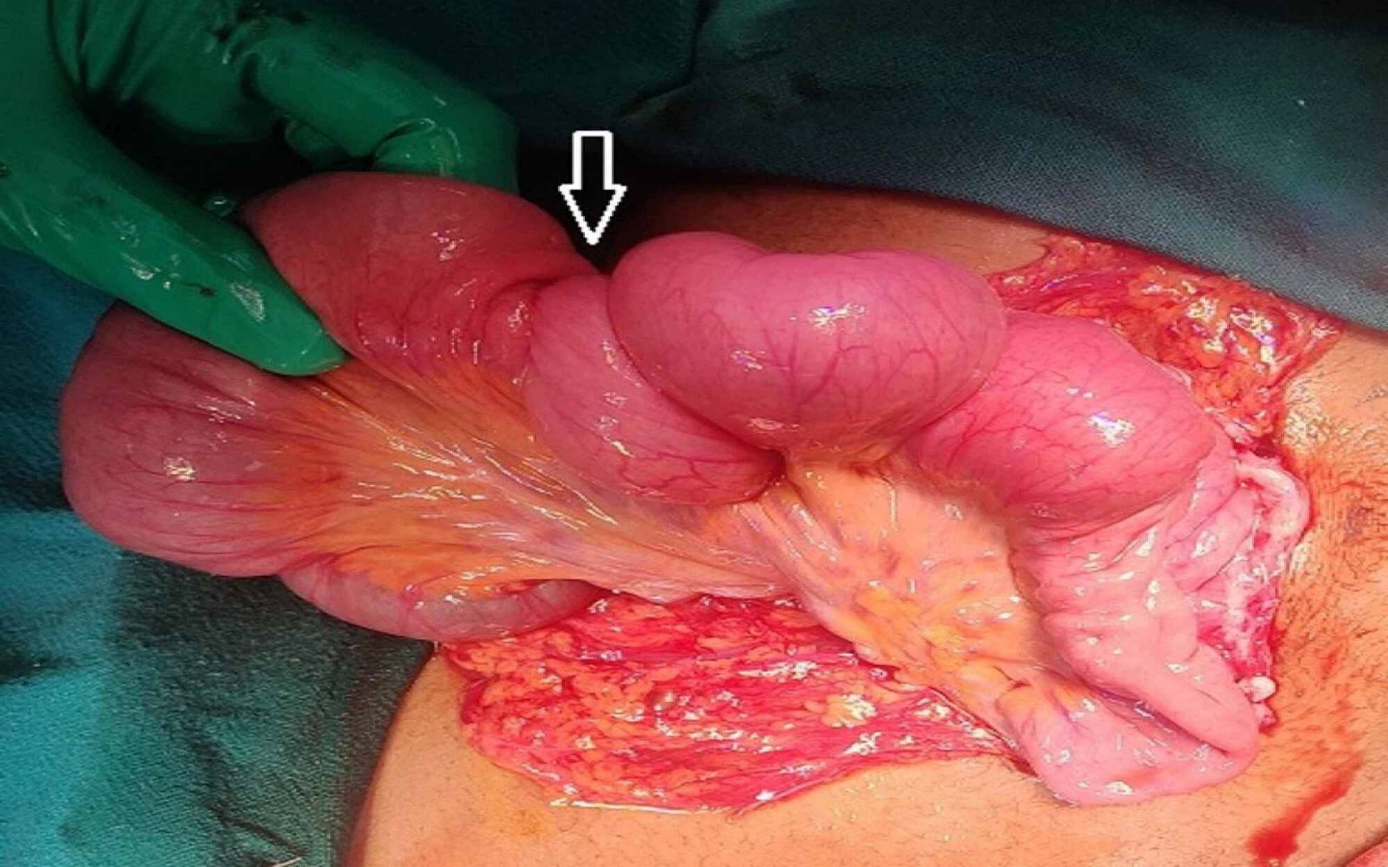
Intraoperative image: The arrow is depicting the site of distal jejunojejunal intussusception where Foley's catheter balloon was found.

Intra-operatively successful reduction of both sites of intussusception was done and the primary cause of distal intussusception was identified to be the hyper-inflated balloon of Foley’s catheter (Figure 3). The likely cause of proximal intussusception was the recurrent push of proximal bowel against lax and crumpled distal bowel leading to its invagination. Reinsertion of FJ tube from a different site with Ryle’s tube was done and attached to the anterior abdominal wall. The patient had no complaints postoperatively and had a normal postoperative stay in hospital.

## Discussion

FJ is a surgical procedure to pursue enteral feeding as a method for nutritional support to a patient due to any cause for which oral intake is not possible [3]. Various complications associated with jejunostomy tube are listed in Table 1 [4]. They are seen more commonly in men and young infants and with the presence of distal pigtail on the tube [2,5].

**Table 1:** Complications of feeding jejunostomy tube

Type	Complications
Mechanical	Coiling, kinking, knotting, mal-positioning, retrograde flow, occlusion of the tube
Infectious	Wound infection, peritubal biliary leakage
Gastrointestinal	Small-bowel obstruction, non-obstructive small-bowel narrowing, extra-luminal tracks or collections, jejunal hematomas, intussusceptions
Metabolic	Hyperglycemia, hypokalemia, water and electrolyte imbalance, hypophosphatemia, and hypomagnesemia

On the basis of severity, the complications are divided based on time of presentation into early (<30 days) or late (>30 days) [6], majority of them being minor. Minor complications can be reduced by using proper technique for insertion of tube followed by meticulous postoperative management. Regular follow-up of the patient also reduces the incidence of nutrition-related complications [3]. Among these various complications, JTI is rarest and has an incidence of one per cent [7,8]. Its diagnosis is difficult as initially it does not interfere with feeding habits of patient and with advanced disease, there are signs and symptoms of obstruction, i.e. vomiting, pain abdomen and inability to pass faeces and flatus [9]. The exact mechanism for occurrence of JTI is unknown; however, many mechanisms have been proposed - tip of the tube acting as a lead point, tube-induced inflammatory reaction causing hypertrophy of the mucosa which can form the lead point, retrograde peristalsis of jejunum due to vomiting and injecting force during feeding and reduced mesenteric fat in poorly built patients which allows free movement of intestine [9]. Our patient was of thin built secondary to decreased intake due to previous corrosive acid intake so there was minimal fat in the abdominal cavity, allowing free movement of bowel loops. Along with the hyper-inflated bulb of the Foley, this hypermobile bowel served as the lead point probably leading to the distal intussusceptions which subsequently led to the formation of proximal intussusception due to shortening of mesentery.

Currently, USG is the initial investigation of choice as it is highly sensitive (98%-100%) for detection of intussusception [10]. The classical signs on USG are “target” or “doughnut” or “pseudokidney” appearance due to hypoechoic outer ring (outer bowel wall) and a hyperechoic centre (inner bowel wall). On axial scan “multiple concentric ring sign” and “crescent-in-doughnut sign” are seen and on longitudinal scan “sandwich sign” and “hayfork sign” are seen.

Cross-sectional imaging is required in cases where the initial imaging modalities are not able to provide the diagnosis and to confirm the diagnosis before taking the patient for definitive surgical management. In emergency, the patient CT is the imaging modality of choice. The ominous signs on CT include non-enhancement of the wall of intussusceptum and detection of associated lead point. In our patient, CT identified the entire extent of proximal site of intussusception. It confirmed mucosal enhancement of intussusceptum at distal site with edematous bowel wall confirming viability and irreducibility.

Surgical treatment is indicated if the patient is in unresponsive shock, the intussusception is irreducible or on imaging a lead point or necrosis or perforation is present [10]. If bowel is non-ischemic, it can be managed with surgical reduction but requires a resection if there is gangrenous change, perforation or stenosis. In our patient, exploratory laparotomy was undertaken as the imaging was suggestive of a long length of intussusceptum with irreducible intussusception at the distal site and impending gangrene. Intraoperatively, both the proximal and distal sites of intussusception were identified without any gangrenous change. The hyper-inflated Foley’s balloon was confirmed as the lead point for the distal intussusception. Further, the repeated push of Foley’s balloon distally by peristalsis caused the crumbling of bowel between FJ entry point and Foley’s balloon over a fixed length of catheter and led to another intussusception proximally. The patient was treated with operative reduction without resection of small intestine. She was well postoperatively.

The learning points from the present case are that one may avoid the use of Foley’s catheter as a feeding tube. Second, even if it has been used, the balloon of Fley’s catheter should not be kept inflated for a long time. Lastly, surgeons should keep intussusception as one of the differentials of complications pertaining to FJ especially when the patient presents with obstructive symptoms.

## Conclusions

Jejunostomy tube-induced intussusception is an uncommon complication after FJ but should be diligently worked up if a patient presents with sudden onset pain abdomen especially 3-4 weeks after the procedure with symptoms of small bowel obstruction. Proper operative case history and evaluation of the case file is essential in these patients to see what tube has been used for FJ before undertaking surgery.
